# Total knee arthroplasty in the outpatient vs inpatient settings: impact of site of care on early postoperative economic and clinical outcomes

**DOI:** 10.1186/s13018-023-03750-4

**Published:** 2023-04-04

**Authors:** Jack Mantel, Jill W. Ruppenkamp, Maricruz Cantu, Chantal E. Holy

**Affiliations:** 1 Health Economics and Market Access, DePuy Synthes, Leeds, UK; 2 Epidemiology and Real-World Data Sciences, Johnson and Johnson MedTech, 410 George Street, New Brunswick, NJ 08933 USA; 3 Health Economics and Market Access, Joint Reconstruction, DePuy Synthes, 700 Orthopedic Drive, Warsaw, IN 46582 USA

**Keywords:** Total knee replacement, Outpatient care, Outcomes research, Health care cost, Health resources

## Abstract

**Background:**

The incidence of total knee arthroplasty (TKA) surgery performed in the outpatient setting has increased as a result of improved perioperative recovery protocols, bundled payments, and challenges brought by the coronavirus disease of 2019 (COVID-19) pandemic on health systems. This study evaluates early postoperative clinical and economic outcomes of patients treated in the inpatient vs outpatient setting using the Attune Knee System (AKS).

**Methods:**

Patients with an elective, primary TKA implanted with the AKS, from Q4 2015 to Q1 2021, were identified within the Premier Healthcare Database. The index was defined as the admission date for inpatient cases and the service day for outpatient procedures. Inpatient and outpatient cases were matched on patient characteristics. Outcomes included 90-day all-cause readmissions, 90-day knee reoperations, and index- and 90-day costs of care. Generalized linear models were used to evaluate outcomes (Reoperation: binomial distribution; costs: Gamma distribution with log link).

**Results:**

Before matching, 39,337 inpatient and 9,365 outpatient cases were identified, with greater comorbidities in the inpatient cohort. The outpatient cohort had a lower average Elixhauser Index (EI) compared to the inpatient cohort (1.94 (standard deviation (SD): 1.46) vs 2.17 (SD: 1.53), p < 0.001), and the rates for each individual comorbidities were also slightly lower in the outpatient compared to the inpatient cohorts. Post-match, 9,060 patients were retained in each cohort [mean age: ~ 67, EI = 1.9 (SD: 1.5), 40% male]. Post-match comorbidity rates were similar between inpatient and outpatient cohorts (outpatient EI: 1.94 (SD: 1.44)–inpatient EI: 1.96 (SD: 1.45), p = 0.3516): in both, 54.1% of patients had an EI between 1 and 2, and 5.1% had an EI ≥ 5. No differences were observed in 3-month reoperation rates (0.6% in outpatient, 0.7% in inpatient cohort). Index and post-index 90-day costs were lower in the outpatient vs inpatient cases [(savings for index-only costs: $2,295 (95% CI: $1,977–$2,614); 90 days post-index knee-related care only: $2,540 (95% CI: $2,205–$2,876); 90 days post-index all-cause care: $2,679 (95% CI: $2,322–$3,036)].

**Conclusions:**

Compared to matched inpatient cases, outpatient TKA cases treated with AKS showed similar 90-day outcomes, at lower cost.

**Supplementary Information:**

The online version contains supplementary material available at 10.1186/s13018-023-03750-4.

## Background

In the last 20 years, the US healthcare system demonstrated increasing trends toward outpatient care delivery. This transition of patient care from inpatient to outpatient setting made it more accessible and affordable for many healthcare consumers. Patients benefited from cheaper services provided at nearby outpatient facilities [1].

The total knee arthroplasty (TKA) procedure has become one of the outstanding examples of orthopedic surgeries being increasingly performed at outpatient settings. On January 1, 2018, the Center for Medicaid and Medicare Services made a policy change removing TKA as an inpatient-only procedure and opening the opportunity for reimbursement for outpatient TKA in the Medicare population [2, 3]. This resulted in 15% shift of TKA procedures to outpatient facilities in Florida hospitals within one year after Medicare’s decision. Meanwhile, the volume of outpatient privately insured TKA cases doubled and reached to 25% [3].

The policy change was essential for addressing increased demand of TKA procedure. Thus far, there have been observed growing trends in the incidence of TKA and a move to younger ages [4, 5]. The US National Inpatient Sample also suggests increased use of TKA in coming years [6, 7]. On the bases of 2000-to-2014 data, growth in primary TKA may reach 56%, 110%, 182% and 401% in 2020, 2025, 2030, and 2040, respectively, representing a total of 1.06, 1.27, 1.92 and 3.42 million procedures [6].

Since 2020, the coronavirus disease of 2019 (COVID-19) pandemic has also increased the incidence of outpatient TKA, as inpatient beds and capacities were reserved for COVID-19 sufferers. The impact of the pandemic reinforced trends already in place due to value-based and bundled payment initiatives, and constant pressures on hospital costs [2, 8].

Preliminary analyses of outcomes in patients treated for TKA in the outpatient vs the inpatient setting suggested that the outpatient setting does not increase risk for readmissions or complications [9–11]. A systematic review and meta-analysis of inpatient vs outpatient total joint replacement cases also concluded that complication rates were similar for both sites of care [12].

Key limitations to greater adoption of outpatient TKA include: (1) patient perception: early data suggest patients may not feel safe undergoing TKA in the outpatient setting, as they worry about pain and mobility [13]; and (2) patient selection: not all patients may be suited for outpatient care, and careful patient selection may be required [11, 14, 15].

To the best of our knowledge, studies evaluating inpatient vs outpatient TKA care have been brand-agnostic, thus including various surgical approaches and implant types, which may add heterogeneity or bias in the analyses. This study was therefore designed to evaluate outcomes of patients treated in the outpatient vs inpatient setting for TKA using only one brand, the Attune Knee System (AKS). The AKS is a total knee replacement implant designed to improve stability and motion of the knee joint. The AKS was launched in the USA in 2011 and was upgraded by two new technologies in 2014. The advanced components (rotating platform design and anatomic patella) provided improved range of motion and reduced implant wear of this device [16]. The prior research and registries have demonstrated favorable clinical outcomes of patients treated with AKS in the inpatient care [17, 18]. This study provided further assessments of the patients’ outcomes selected for outpatient care, as compared to those treated for inpatient care with AKS.

## Methods

### Database

Our retrospective cohort study was conducted in the PREMIER Healthcare database (PHD). The PHD contains complete clinical coding, including diagnosis, procedures, and hospital-prescribed medications from more than 20% of all hospital admissions throughout the United States (> 1040 hospitals and hospital systems). Although the database excludes federally funded hospitals (e.g., Veterans Affairs), the hospitals included are nationally representative based on bed size, geographic region, location (urban/rural) and teaching hospital status. The database contains a date-stamped log of all billed items by cost-accounting department including medications; laboratory, diagnostic, and therapeutic services; and primary and secondary diagnoses for each patient’s hospitalization. Identifier-linked enrollment files provide demographic and payor information. Detailed service level information for each hospital day is recorded; this includes details on medication and devices received.

### Cohort

The study cohort included adult patients with an elective TKA with the Attune Knee System (DePuy Synthes, Warsaw, IN) performed from Q4 2015 to Q1 2021. The Q4 2015 date was selected as this represents the start of the international classification of disease (ICD)-10 era and ensures consistent coding of cases across all patients. The groups considered herein were patients treated in the in- vs outpatient setting. Index was defined as the admission date for the TKA surgery for inpatient cases, equivalent to service day for same-day or outpatient procedures. Providers of included patients had continuous data availability in PHD for at least 90 days post-index, to allow assessment of 90-day outcomes. Patients were excluded from the study if they met the following criteria: age < 18 years, diagnosis of knee fracture, cancer, aseptic loosening, infection or osteomyelitis present at index admission (to ensure that no revision case was accidently included in the cohort). Patients were excluded from the analyses if they had a procedure code indicative of a unicondylar knee procedure at time of index, or bilateral TKAs during the same admission (or within 90 days of index for 90-day outcomes).

### Outcomes

Outcomes included 90-day knee-related patient costs. All costs analyzed in our study refer to hospital costs, not payor payments, and thus show the impact of care on the provider specifically. Costs were subcategorized as knee-related vs all-cause. All-cause included all reported costs. Knee-related included all costs with a knee- or postoperative care or complication procedure or diagnosis. Additional outcomes included: index and all-cause 90-day costs, reoperations and all-cause 90-day readmissions after TKA, home discharge, operating room time and length of hospital stay.

### Variables

Patient variables included patient characteristics (age, gender, race, marital status, payor category, discharge status, body mass index (BMI) category), provider characteristics (urban or rural, beds group, region, division, physician specialty) and patient chronic comorbidities at time of index admission, specifically all 31 comorbidities from the Elixhauser Index (EI).

### Statistical analyses

Descriptive statistics were reported for all study variables and outcomes, for each of the cohorts. Means, median, interquartile ranges, minimum and maximum and standard deviations were reported for continuous variables and frequencies and percentages were reported for categorical variables. Standardized mean differences (SMD) for all variables were assessed between the inpatient and the outpatient cohorts, to identify variables with greatest variability between the groups. To adjust for confounding, direct and propensity score matching were used, focusing on variables with greater SMD between unmatched cohorts and variables known to affect outcomes. Patients with outpatient surgery were matched 1:1 with the inpatient cohort, on patient age category, sex, Elixhauser category and hospital size. Individual comorbidities were matched using propensity scores. Nearest neighbor matching with calipers of width equal to 0.1 of the pooled standard deviation of the logit of the propensity score was used. The covariates not balanced in propensity score matching (PSM) were controlled for in Generalized Linear Models (GLM). Crude and adjusted costs were calculated at index and 90 days. Costs were modeled using GLM with log link and gamma distributions. Costs were analyzed for all inpatient vs outpatient cohorts, as well as by payor type (commercial, Medicare and Medicaid). The impact of inpatient vs outpatient care, payor, race and gender on total costs, discharge to SNF, and rates of readmissions and reoperations were analyzed using logistic regression models.

## Results

There were 9,365 outpatient and 39,337 inpatient cases included in the study cohort. Patient characteristics (baseline demographics, EI score and the 31 EI comorbidities) and hospital characteristics, before and after matching, are shown in Tables 1 and 2, respectively.

**Table 1:** Patient and provider characteristics of patients treated with TKA, in the in- vs. outpatient setting, before and after matching

Variable	Pre-Match			Post-Match		
	Outpatient	Inpatient	SMD	Outpatient	Inpatient	SMD
N	9,365	39,337		9,060	9,060	
Age: mean (SD)	67.2 (8.7)	66.9 (9.4)	0.0390	67.4 (8.4)	67.5 (8.6)	0.0120
Age Category			0.1430			0.0000
18 to 34	2 (0.0%)	46 (0.1%)		0	0	0.0000
35 to 44	75 (0.8%)	383 (1.0%)		43 (0.5%)	43 (0.5%)	
45 to 54	656 (7.0%)	3,487 (8.9%)		592 (6.5%)	592 (6.5%)	
55 to 64	2,445 (26.1%)	11,227 (28.5%)		2,369 (26.1%)	2,369 (26.1%)	
65 to 74	4,346 (46.4%)	15,654 (39.8%)		4,264 (47.1%)	4,264 (47.1%)	
75 and Older	1,841 (19.7%)	8,540 (21.7%)		1,792 (19.8%)	1,792 (19.8%)	
Sex: Male (vs Female)	3,787 (40.4%)	15,383 (39.1%)	0.0270	3,648 (40.3%)	3,648 (40.3%)	0.0000
Race Category			0.0760			0.0760
Asian	82 (0.9%)	382 (1.0%)		82 (0.9%)	85 (0.9%)	
Black	718 (7.7%)	3,195 (8.1%)		694 (7.7%)	651 (7.2%)	
Other	546 (5.8%)	2,738 (7.0%)		525 (5.8%)	602 (6.6%)	
Unknown	143 (1.5%)	354 (0.9%)		135 (1.5%)	72 (0.8%)	
White	7,876 (84.1%)	32,668 (83.0%)		7,624 (84.2%)	7,650 (84.4%)	
Payor			0.1130			0.0700
Commercial	2,622 (28.0%)	12,763 (32.4%)		2,547 (28.1%)	2,436 (26.9%)	
Medicaid	296 (3.2%)	1,307 (3.3%)		230 (2.5%)	236 (2.6%)	
Medicare	6,146 (65.6%)	23,707 (60.3%)		6,043 (66.7%)	6,041 (66.7%)	
Other	301 (3.2%)	1,560 (4.0%)		240 (2.6%)	347 (3.8%)	
Urban Hospital (vs Rural)	7,870 (84.0%)	34,588 (87.9%)	0.1120	7,700 (85.0%)	7,657 (84.5%)	0.0130
Hospital Size			0.3140			0.0000
000 to 099	1,449 (15.5%)	4,113 (10.5%)		1,367 (15.1%)	1,367 (15.1%)	
100 to 199	1,997 (21.3%)	6,919 (17.6%)		1,912 (21.1%)	1,912 (21.1%)	
200 to 299	1,987 (21.2%)	9,304 (23.7%)		1,957 (21.6%)	1,957 (21.6%)	
300 to 399	1,896 (20.2%)	8,082 (20.5%)		1,865 (20.6%)	1,865 (20.6%)	
400 to 499	1,310 (14.0%)	4,480 (11.4%)		1,245 (13.7%)	1,245 (13.7%)	
500 or larger	726 (7.8%)	6,439 (16.4%)		714 (7.9%)	714 (7.9%)	

*SD* standard deviation, *SMD* standardized mean differences, *TKA* total knee arthroplasty

**Table 2:** Comorbidity of patients in the in- and outpatient TKA cohorts, before and after matching

Variable	Pre-Match			Post-Match		
	Outpatient	Inpatient	SMD	Outpatient	Inpatient	SMD
Elixhauser Index: mean (SD)	1.94 (1.46)	2.17 (1.53)	0.1570	1.94 (1.44)	1.96 (1.45)	0.0180
Elixhauser Score Category			0.1490			0.0000
0	1,488 (15.9%)	4,701 (12.0%)		1,388 (15.3%)	1,388 (15.3%)	
1–2	4,989 (53.3%)	20,436 (52.0%)		4,902 (54.1%)	4,902 (54.1%)	
3–4	2,382 (25.4%)	11,240 (28.6%)		2,305 (25.4%)	2,305 (25.4%)	
5 or more	506 (5.4%)	2,960 (7.5%)		465 (5.1%)	465 (5.1%)	
*Individual Comorbidities*						
Hypertension	6,057 (64.7%)	27,169 (69.1%)	0.0930	5,926 (65.4%)	5,953 (65.7%)	0.0060
Diabetes (all types)	1,795 (19.2%)	8,944 (22.7%)	0.0880	1,733 (19.1%)	1,868 (20.6%)	0.0370
Obesity	2,741 (29.3%)	12,154 (30.9%)	0.0360	2,637 (29.1%)	2,439 (26.9%)	0.0490
Hypothyroidism	1,474 (15.7%)	6,743 (17.1%)	0.0380	1,429 (15.8%)	1,431 (15.8%)	0.0010
Chronic Pulmonary Disease	1,324 (14.1%)	6,267 (15.9%)	0.0500	1,264 (14.0%)	1,289 (14.2%)	0.0080
Depression	1,231 (13.1%)	5,998 (15.2%)	0.0600	1,177 (13.0%)	1,242 (13.7%)	0.0210
Cardiac Arrhythmia	838 (8.9%)	4,093 (10.4%)	0.0490	823 (9.1%)	825 (9.1%)	0.0010
Renal failure	621 (6.6%)	2,994 (7.6%)	0.0380	579 (6.4%)	585 (6.5%)	0.0030
Rheumatoid Arthritis and Collagen Diseases	377 (4.0%)	1,847 (4.7%)	0.0330	366 (4.0%)	405 (4.5%)	0.0210
Congestive Heart Failure	327 (3.5%)	1,537 (3.9%)	0.0220	318 (3.5%)	272 (3.0%)	0.0290
Valvular Disease	230 (2.5%)	1,425 (3.6%)	0.0680	224 (2.5%)	297 (3.3%)	0.0480
Peripheral Vascular Disorders	205 (2.2%)	1,038 (2.6%)	0.0290	200 (2.2%)	190 (2.1%)	0.0080
Fluid and electrolyte disorders	197 (2.1%)	1,127 (2.9%)	0.0490	188 (2.1%)	218 (2.4%)	0.0220
Other Neurological Disorders	160 (1.7%)	832 (2.1%)	0.0300	149 (1.6%)	174 (1.9%)	0.0210
Liver disease	126 (1.3%)	534 (1.4%)	0.0010	119 (1.3%)	113 (1.2%)	0.0060
Coagulopathy	100 (1.1%)	665 (1.7%)	0.0530	96 (1.1%)	119 (1.3%)	0.0230
Deficiency Anemia	91 (1.0%)	538 (1.4%)	0.0370	84 (0.9%)	103 (1.1%)	0.0210
Pulmonary Circulatory Disorders	52 (0.6%)	298 (0.8%)	0.0250	51 (0.6%)	67 (0.7%)	0.0220
Alcohol Abuse	57 (0.6%)	317 (0.8%)	0.0240	54 (0.6%)	49 (0.5%)	0.0070
Drug Abuse	56 (0.6%)	468 (1.2%)	0.0630	50 (0.6%)	75 (0.8%)	0.0330
Psychoses	10 (0.1%)	71 (0.2%)	0.0190	8 (0.1%)	17 (0.2%)	0.0270

*SD* standard deviation, *SMD* standardized mean differences, *TKA* total knee arthroplasty

### Baseline demographic characteristics and comorbidities

#### Pre-match cohorts

As shown in Table 1: In the outpatient cohort, the mean age (standard deviation (SD)) of patients was 67.2 (8.7) years, 40.4% were male, most patients were Caucasians (84.1%). Nearly two-thirds of patients had Medicare coverage (65.6%) and most of the remaining had commercial health insurance (28.0%). Patients in the inpatient cohort had mean (SD) overall age of 66.9 (9.4) years, 39.1% were male, and 83.0% were Caucasian. Most of the patients had Medicare coverage (60.3%). 84.0% outpatient and 87.9% inpatient cases were performed in urban hospitals, and only 7.8% outpatient vs 16.4% inpatient cases were performed in a hospital with 500 beds or more The patient demographics, stratified by payor type (Commercial, Medicare vs Medicaid) are shown in the Supplemental File, in Additional file 1: Table S1. Comorbidities were common and are shown in Table 2. The pre-match, outpatient cohort had a slightly lower EI compared to the inpatient cohort (1.94 (SD: 1.46) vs 2.17 (SD: 1.53), p < 0.001), and the rates for each individual comorbidities were also slightly lower in the outpatient compared to the inpatient cohorts. Slightly more than half (53.3%) of outpatient cases had an Elixhauser comorbidity score between 1 and 2 and the most common comorbidities in this cohort were: uncomplicated hypertension (56.5%), chronic pulmonary disease (14.1%), uncomplicated diabetes (14.3%), hypothyroidism (15.7%), and obesity (29.3%). In the inpatient cohort, the Elixhauser comorbidity score ranged from 1 to 2 for 52.0% of patients. Similar to outpatient cohort, the most prevalent comorbidities were uncomplicated hypertension (60.1%), chronic pulmonary disease (15.9%), uncomplicated diabetes (16.4%), hypothyroidism (17.1%) and obesity (30.9%). Comorbidities stratified by payor type are shown in Additional file 1: Table S2.

#### Matching

**Fig. 1:**
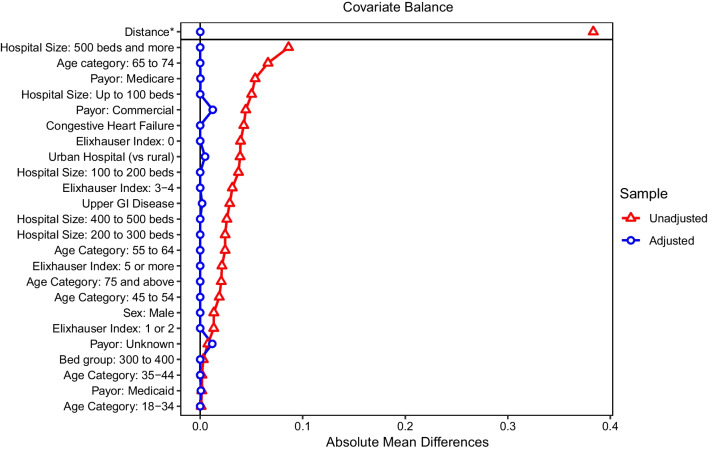
Covariate balance in the initial vs matched dataset. After matching, absolute mean differences were lower than 0.05 for all included covariates. (For all covariates: the x-axis represents absolute mean differences, except for the Distance, for which the x-axis represents the standardized mean difference)

Pre- and post-match SMDs are shown in Fig. 1. For all variables of interest, the final SMD were less than 0.1, suggesting appropriate matching between the inpatient and outpatient cohorts. Post-match, 9,060 patients were retained in the inpatient and outpatient cohorts (96.7% of the outpatient and 23% of the inpatient cohorts, for a 1:1 match).

#### Post-match cohorts

After matching (Table 1 and Additional file 1: Table S1 for stratification by payor), age was comparable across groups (~ 67 years), with 40.3% males, majority white patients (~ 84), majority Medicare (66.7%), and ~ 85% cases performed in the urban setting. In the matched dataset, only ~ 8% of inpatient and outpatient cases were performed in hospitals with 500 beds or larger. As shown by post-match SMD values, cohort differences were addressed by the matching, resulting in SMD values lower than 0.1 for all variables.

As shown in Table 2 and Additional file 1: Table S2: post-match comorbidities were also similar across cohorts (EI for outpatient cohort: 1.94 (SD: 1.44); EI for inpatient cohort: 1.96 (SD: 1.45), p = 0.3516). For both inpatient and outpatient cohorts, 54.1% patients had an Elixhauser score between 1 and 2, and 5.1% had an Elixhauser score 5 or above. All individual comorbidities had balanced for SMD < 0.1. A slight imbalance still existed in obesity rate, with the outpatient cohort having slightly more obese patients (29.1%) versus the inpatient cohort (26.9%).

### Cost analyses

Costs were estimated using GLM models. Modeling was done to address any residual imbalance between groups. As shown in Table 3: Index costs averaged $14,874 (95% CI: $14,665–$15,083) for outpatient cases and $17,170 (95% CI: $16,928–$17,411) for inpatient cases, with a marginal mean difference of $2,295 (95% CI: $1,977–$2,614). Total costs over 90 days are also shown in Table 3. The costs for patients covered by Commercial vs Medicare and Medicaid insurance are shown in Additional file 1: Table S3. Knee-related costs (excluding costs for services that did not carry a knee-related or postoperative related diagnosis or procedure code) and all-cause costs remained statistically lower in the outpatient cohort over 90-day postoperative period.

**Table 3:** Hospital costs of patients treated with inpatient vs outpatient TKA

Index costs	Outpatient	$14,874 (95% CI: $14,665–$15,083)
	Inpatient	$17,170 (95% CI: $16,928–$17,411)
	*Marginal Increase from Outpatient to Inpatient*	*$2,295 (95% CI: $1,977–$2,614)*
90-Day Knee Related Costs*	Outpatient	$15,196 (95% CI: $14,977–$15,415)
	Inpatient	$17,737 (95% CI: $17,481–$17,992)
	*Marginal Increase from Outpatient to Inpatient*	*$2,540 (95% CI: $2,205–$2,876)*
90-Day All-Cause Costs*	Outpatient	$15,718 (95% CI: $15,486–$15,951)
	Inpatient	$18,397 (95% CI: $18,125–$18,669)
	*Marginal Increase from Outpatient to Inpatient*	*$2,679 (95% CI: $2,322–$3,036)*

*Includes index costs

*CI* confidence interval, *TKA* total knee arthroplasty

### Additional endpoints

Additional outcomes in the matched cohorts are shown in Table 4. Whereas p values are shown for these exploratory endpoints, the study was not assessed a-priori for power to evaluate each of these individually. The threshold for significance, using a Bonferroni correction, should be set at 0.00625 (0.05/8). Length of stay was evidently lower in the outpatient vs inpatient cohorts, as outpatient cases did not have any overnight stay. There were differences in discharge destination, which may be due to confounding variables related to patients in outpatient care more likely to have home support vs patients in inpatient care, and SNF discharge requiring a minimum number of inpatient days for Medicare patients. Our analysis could not take social and home support variables into consideration, as these would not be available in the database. A similar explanation might relate to the all-cause readmission, which was higher in the inpatient vs outpatient, whereas reoperation rates were not different. A small difference in operating room (OR) time was observed. Whereas this difference was statistically significant, it was not clinically meaningful (less than 2 min). Similar to Table 4, analysis of exploratory endpoints by payor type is shown in Additional file 1: Table S4.

**Table 4:** Descriptive statistics of exploratory outcomes following inpatient vs outpatient TKA, in the matched cohorts

	Outpatient	Inpatient	*P* value*
Length of Stay: mean days (SD)	0.00 (0.16)	2.08 (1.20)	< 0.001
Discharge status			< 0.001
Discharged to HHO	1,639 (18.1%)	3,404 (37.6%)	
Home	7,173 (79.2%)	4,289 (47.3%)	
SNF/Other	248 (2.7%)	1,367 (15.1%)	
Operating Room Time: mean minutes (SD)	132.10 (34.30)	133.83 (35.72)	0.001
Mean Costs: US (SD)**			
Index admission	14,863 (5,386)	17,151 (15,959)	< 0.001
Index with 90 days post-index	15,707 (6,917)	18,388 (17,278)	< 0.001
Index with 90 days post-index, knee related	15,182 (6,014)	17,725 (16,720)	< 0.001
Reoperation at 90 Days: N (%)	55 (0.6%)	62 (0.7%)	0.578
All-Cause Readmissions at 90 Days: N (%)	2,543 (28.1%)	2,884 (31.8%)	< 0.001

*SD* standard deviation, *TKA* total knee arthroplasty

*The threshold for significance, using a Bonferroni correction, is set at 0.006

**Does not include SNF costs

### Impact of patient and provider characteristics on SNF discharge, costs, readmission and reoperation rates

Logistic regression models were built to evaluate the impact of patient and provider characteristics on risk of SNF discharge, costs, and risks of reoperation and readmissions. The outputs from the models are shown in the supplemental files Additional file 1: Tables S5–S10. For all costs analyzed herein, inpatient index (vs outpatient) was associated with increased cost. For index hospital costs (Additional file 1: Table S5), payor and sex did not significantly increase costs; however, smaller hospitals (less than 200 beds, compared to 500 beds and above) had greater costs (6% for hospitals size 0–99 beds, 10% for hospitals size 100–199 beds), as well as hospitals in some US regions (Pacific (37%) and South Atlantic (35%) versus East North Central). Black race (vs White) was associated with increased cost (7%). For all-cause 90-day costs (Additional file 1: Table S6), patient payor and sex did not affect costs, Black race (vs White) was associated with increased costs, and from a provider standpoint, smaller and rural hospitals, and hospitals in Pacific, West South Central and South Atlantic regions, had higher costs. For the knee-related 90-day costs, race did not affect outcomes (Additional file 1: Table S7). For knee-related 90-day costs, only hospital size (smaller hospitals having higher costs), hospital location (rural vs urban) and geographic location were associated with increased costs. Patient characteristics (sex, race, payor) did not affect 90-day knee-related costs.

Odds of discharge to SNF (versus home or home health) based on patient and provider characteristics are shown in Additional file 1: Table S8. Index inpatient (vs outpatient) care was associated with greater odds for SNF discharge (13%). From a provider standpoint, as observed with costs, smaller hospitals, and hospitals in some geographic areas had higher odds of SNF discharge. From a patient characteristics standpoint: patients with Medicare (vs commercial insurance), female patients (vs males) and Black patients (vs White) were at increased risk for SNF discharge.

Odds of reoperation based on patient and provider characteristics are shown in Additional file 1: Table S9. None of the provider variables affected odds of knee reoperation. Inpatient care at index (vs outpatient) was also not associated with greater reoperation odds. The only variable that had a very small potential increase in odds of reoperation was patient payor: patients covered with Medicaid showed a very slight increase in odds of reoperations (1.01, 95% CI: 1.00–1.02, p = 0.02).

Odds of all-cause readmissions, however, were associated with some of the variables analyzed in our study, as shown in Additional file 1: Table S10. From a provider standpoint, smaller, rural hospitals, and East North Central hospitals, were associated with greater all-cause readmissions. Inpatient (vs outpatient) care was also associated with greater odds of all-cause readmission. Patients covered with Medicare and Medicaid (vs commercial), as well as Black patients (vs White), were at increased odds of all-cause readmission.

## Discussion

We used a nationally representative database to evaluate outcomes of elective TKA patients treated with AKS in the outpatient vs inpatient settings. We identified 48,702 patients in a more than 6-year time frame, of which 9,365 patients were outpatient cases. To address patient differences that may be due to selection biases for in- vs outpatient care, we did a thorough 1:1 match, with exact matching on key demographic and comorbid variables, and a PSM on additional, less critical variables, identified as having significant imbalance in the pre-match cohort. Our matched analyses showed that patients treated in the inpatient vs outpatient setting had similar reoperation and readmission rates, with outpatient cases incurring evidently lower costs.

Risk of selection bias is important in outcomes analyses of patients treated in the in- vs outpatient setting, as specific comorbidities may disqualify patients for outpatient surgery. Patient selection has recently been discussed in a 3,015 outpatient case study. In this study, patients with dependent functional status, hypertension, chronic obstructive pulmonary disease, and prolonged operative time were at increased risk of readmission and were at benefit of inpatient care [15]. Body mass index (BMI), age and surgery start time were also associated with inpatient care (versus same-day discharge) [19]. We observed in our pre-match cohort that patients selected for outpatient care had lower EI, indicative of fewer comorbidities, but differences in the rates of each individual comorbidities were small, none of them reached an SMD > 0.1. Interestingly, however, before matching, the outpatient cohort also included severely comorbid patients (5.4% with EI of 5 or greater, 29% obese). To ensure a meaningful comparison, however, we matched our cohorts to ensure no residual imbalance and observed similar outcomes with lower costs in the outpatient cohort vs inpatient cohort.

Our study also identified risk factors for increased costs, SNF discharge or readmission/reoperation rates. Hospital characteristics, such as size and rural status, were associated with increased costs. This may be related to larger hospitals being able to realize economies of scales unavailable to smaller institutions. Independently of size and rural status, geographic location also affected costs, reflecting geographic differences in healthcare delivery. From a patient characteristics standpoint, patients covered with Medicare had greater odds of discharge to SNF, and Medicaid patients had a very slight increase in reoperation rates, but otherwise no differences in hospital costs across payor type. We also observed increased hospital costs, SNF discharge odds and readmission rates in Black vs White patients. We cannot explain these differences, they may be due to unmeasured confounders such as availability of home care, unmeasured morbidities, or other social determinants of health not captured in the databases. Sex did not affect most of the outcomes we analyzed, except for SNF discharge: males were slightly less likely to be discharged to a SNF compared to females (Odds ratio: 0.97, 95% CI: 0.96–0.98).

Our study has the following limitations: the main limitation of our work is that patients that were scheduled for outpatient care, but ended up being admitted, were not identifiable and would simply be listed as inpatient cases. We also could not easily explain why more patients from the inpatient cohort (vs outpatient cohort), in the matched cohorts, were discharged to skilled nursing facilities (SNF) and other higher acuity care settings and had more all-cause readmissions. These findings may be due to confounders such as availability of home support, presence of an able spouse or family members, or general motivation and activity level. Outpatient care may be selected and better suited for highly motivated patients with some independence and support at home, whereas inpatient care may be the default when such support is missing. These confounders would not be analyzable in the database as only diagnoses, procedures, comorbidities and hospital variables could be controlled. These metrics may therefore have a subjective component that other, possibly more objective variables, such as knee-related reoperations, do not. Our study did show that, in patients carefully matched on comorbidities and other key clinical variables, knee reoperation rates were similar in patients with inpatient vs outpatient care.

An additional limitation is related to our database: our study uses data from a hospital database that was not prospectively designed for our study’s purpose. All outcomes and costs were captured from the database and corresponding codes. Miscoding or missing services would not be identifiable.

## Conclusions

Our study showed that patients treated in the outpatient setting had similar outcomes to patients treated in the inpatient setting, after matching on the outpatient cohort’s comorbidities and demographics. We also showed that today’s outpatient cases include patients with severe comorbidities, as seen in the inpatient cohorts.

## Supplementary Information

**Additional file 1**. Baseline characteristics and study results stratified by payor type.

## Data Availability

The data for these analyses were made available to the authors by third-party licenses from Premier, a data provider in the USA. Under the licensing agreement, the authors cannot provide raw data themselves. Other researchers could access the data by purchase through Premier (at: https://premierinc.com/solutions/data-analytics), and the inclusion and exclusion criteria specified in the Methods section would allow them to identify the same cohort of patients we used for these analyses.

## Abbreviations

AKS: Attune Knee System
BMI: Body mass index
COVID-19: Coronavirus Disease of 2019
EI: Elixhauser Index
ICD: International Classification of Disease
GLM: Generalized linear model
OR: Operating room
PHD: Premier Healthcare Database
PSM: Propensity score matched
SD: Standard Deviation
SMD: Standardized mean differences
SNF: Skilled nursing facilities
TKA: Total Knee Arthroplasty

## References

[1] Kacik A. Number of outpatient facilities surges as industry values more convenient, affordable care Modern HealthcareDecember 20, 2018 [Available from: https://www.modernhealthcare.com/article/20181220/NEWS/181229992/number-of-outpatient-facilities-surges-as-industry-values-more-convenient-affordable-care.

[2] Edwards PK, Milles JL, Stambough JB, Barnes CL, Mears SC (2019). Inpatient versus outpatient total knee arthroplasty. J Knee Surg.

[3] Richards MR, Seward JA, Whaley CM (2021). Removing Medicare's outpatient ban and Medicare and private surgical trends. Am J Manag Care.

[4] Maradit Kremers H, Larson DR, Crowson CS, Kremers WK, Washington RE, Steiner CA (2015). Prevalence of total hip and knee replacement in the USA. J Bone Joint Surg Am.

[5] Cram P, Lu X, Kates SL, Singh JA, Li Y, Wolf BR (2012). Total knee arthroplasty volume, utilization, and outcomes among Medicare beneficiaries, 1991–2010. JAMA.

[6] Singh JA, Yu S, Chen L, Cleveland JD (2019). Rates of total joint replacement in the USA: future projections to 2020–2040 using the national inpatient sample. J Rheumatol.

[7] Sloan M, Premkumar A, Sheth NP (2018). Projected volume of primary total joint arthroplasty in the U.S., 2014 to 2030. J Bone Joint Surg Am.

[8] Cherry A, Montgomery S, Brillantes J, Osborne T, Khoshbin A, Daniels T (2021). Converting hip and knee arthroplasty cases to same-day surgery due to COVID-19. Bone Jt Open.

[9] Darrith B, Frisch NB, Tetreault MW, Fice MP, Culvern CN, Della Valle CJ (2019). Inpatient versus outpatient arthroplasty: a single-surgeon, matched cohort analysis of 90-day complications. J Arthroplasty.

[10] Gromov K, Jorgensen CC, Petersen PB, Kjaersgaard-Andersen P, Revald P, Troelsen A (2019). Complications and readmissions following outpatient total hip and knee arthroplasty: a prospective 2-center study with matched controls. Acta Orthop.

[11] Mariorenzi M, Levins J, Marcaccio S, Orfanos A, Cohen E (2020). Outpatient total joint arthroplasty: a review of the current stance and future direction. R I Med J.

[12] Xu J, Cao JY, Chaggar GS, Negus JJ (2020). Comparison of outpatient versus inpatient total hip and knee arthroplasty: a systematic review and meta-analysis of complications. J Orthop.

[13] Adelani MA, Barrack RL (2019). Patient perceptions of the safety of outpatient total knee arthroplasty. J Arthroplasty.

[14] Cassard X, Garnault V, Corin B, Claverie D, Murgier J (2018). Outpatient total knee arthroplasty: readmission and complication rates on day 30 in 61 patients. Orthop Traumatol Surg Res.

[15] Bovonratwet P, Shen TS, Ast MP, Mayman DJ, Haas SB, Su EP (2020). Reasons and risk factors for 30-day readmission after outpatient total knee arthroplasty: a review of 3015 cases. J Arthroplasty.

[16] drugwatch. DePuy Attune Knee Replacements [updated September 24, 2021. Available from: https://www.drugwatch.com/knee-replacement/depuy-attune/#:~:text=The%20DePuy%20Attune%20knee%20system,to%20improve%20stability%20and%20motion.&text=DePuy's%20rotating%20platform%20design%20is,and%20wear%20on%20the%20implant.

[17] Hauer G, Horlesberger N, Klim S, Bernhardt GA, Leitner L, Glehr M (2021). Mid-term results show no significant difference in postoperative clinical outcome, pain and range of motion between a well-established total knee arthroplasty design and its successor: a prospective, randomized, controlled trial. Knee Surg Sports Traumatol Arthrosc.

[18] Moorthy V, Lai MC, Liow MHL, Chen JY, Pang HN, Chia SL (2021). Similar postoperative outcomes after total knee arthroplasty with measured resection and gap balancing techniques using a contemporary knee system: a randomized controlled trial. Knee Surg Sports Traumatol Arthrosc.

[19] Moore MG, Brigati DP, Crijns TJ, Vetter TR, Schultz WR, Bozic KJ (2020). Enhanced selection of candidates for same-day and outpatient total knee arthroplasty. J Arthroplasty.

