# Supplementary material for: Total knee arthroplasty in the outpatient vs inpatient settings: impact of site of care on early postoperative economic and clinical outcomes
Source: J Orthop Surg Res. 2023 Apr 4;18:273. doi: 10.1186/s13018-023-03750-4 (PMC10071243; doi:10.1186/s13018-023-03750-4)
Supplement: Supplementary file 1 — Additional file 1. Baseline characteristics and study results stratified by payor type. [file 13018_2023_3750_MOESM1_ESM.docx]

**Table S1: Patient and hospital characteristics, by admission and payer type, before and after matching.**

|  | PreMatch | | | | | | Postmatch | | | | | |
| --- | --- | --- | --- | --- | --- | --- | --- | --- | --- | --- | --- | --- |
|  | Commercially-Insured | | Medicare | | Medicaid | | Commercially-Insured | | Medicare | | Medicaid | |
|  | Outpatient | Inpatient | Outpatient | Inpatient | Outpatient | Inpatient | Outpatient | Inpatient | Outpatient | Inpatient | Outpatient | Inpatient |
| N | 2,622 | 12,763 | 6,146 | 23,707 | 296 | 1,307 | 2,547 | 2,436 | 6,043 | 6,041 | 230 | 236 |
| Age : mean (SD) | 59.78 (6.42) | 59.40 (6.72) | 71.22 (6.46) | 71.86 (7.18) | 56.59 (6.71) | 57.04 (8.61) | 59.69 (6.21) | 59.73 (6.32) | 71.32 (6.27) | 71.48 (6.48) | 57.17 (5.80) | 56.61 (6.06) |
| Age Category |  |  |  |  |  |  |  |  |  |  |  |  |
| 18 to 34 | 1 (0.0%) | 23 (0.2%) | 0 (0.0%) | 7 (0.0%) | 1 (0.3%) | 11 (0.8%) | 0 (0.0%) | 0 (0.0%) | 0 (0.0%) | 0 (0.0%) | 0 (0.0%) | 0 (0.0%) |
| 35 to 44 | 38 (1.4%) | 223 (1.7%) | 11 (0.2%) | 49 (0.2%) | 16 (5.4%) | 73 (5.6%) | 34 (1.3%) | 34 (1.4%) | 1 (0.0%) | 1 (0.0%) | 5 (2.2%) | 5 (2.1%) |
| 45 to54 | 447 (17.0%) | 2,397 (18.8%) | 71 (1.2%) | 408 (1.7%) | 82 (27.7%) | 393 (30.1%) | 432 (17.0%) | 404 (16.6%) | 58 (1.0%) | 57 (0.9%) | 61 (26.5%) | 64 (27.1%) |
| 55 to64 | 1,666 (63.5%) | 8,034 (62.9%) | 444 (7.2%) | 1,710 (7.2%) | 184 (62.2%) | 666 (51.0%) | 1,649 (64.7%) | 1,591 (65.3%) | 423 (7.0%) | 422 (7.0%) | 158 (68.7%) | 161 (68.2%) |
| 65 to74 | 426 (16.2%) | 1,859 (14.6%) | 3,845 (62.6%) | 13,335 (56.2%) | 11 (3.7%) | 115 (8.8%) | 400 (15.7%) | 380 (15.6%) | 3,809 (63.0%) | 3,809 (63.1%) | 5 (2.2%) | 5 (2.1%) |
| 75 and above | 44 (1.7%) | 227 (1.8%) | 1,775 (28.9%) | 8,198 (34.6%) | 2 (0.7%) | 49 (3.7%) | 32 (1.3%) | 27 (1.1%) | 1,752 (29.0%) | 1,752 (29.0%) | 1 (0.4%) | 1 (0.4%) |
| Sex: Male (vs Female) | 1,126 (42.9%) | 5,408 (42.4%) | 2,394 (39.0%) | 8,605 (36.3%) | 95 (32.1%) | 428 (32.7%) | 1,091 (42.8%) | 1,050 (43.1%) | 2,343 (38.8%) | 2,343 (38.8%) | 71 (30.9%) | 72 (30.5%) |
| Race Category |  |  |  |  |  |  |  |  |  |  |  |  |
| Asian | 25 (1.0%) | 132 (1.0%) | 52 (0.8%) | 216 (0.9%) | 3 (1.0%) | 17 (1.3%) | 25 (1.0%) | 21 (0.9%) | 52 (0.9%) | 54 (0.9%) | 3 (1.3%) | 3 (1.3%) |
| Black | 249 (9.5%) | 1,090 (8.5%) | 381 (6.2%) | 1,633 (6.9%) | 54 (18.2%) | 259 (19.8%) | 247 (9.7%) | 192 (7.9%) | 371 (6.1%) | 361 (6.0%) | 46 (20.0%) | 49 (20.8%) |
| Other | 224 (8.5%) | 912 (7.1%) | 284 (4.6%) | 1,551 (6.5%) | 18 (6.1%) | 169 (12.9%) | 218 (8.6%) | 176 (7.2%) | 279 (4.6%) | 369 (6.1%) | 14 (6.1%) | 27 (11.4%) |
| Unknown | 37 (1.4%) | 103 (0.8%) | 89 (1.4%) | 203 (0.9%) | 4 (1.4%) | 26 (2.0%) | 36 (1.4%) | 19 (0.8%) | 89 (1.5%) | 44 (0.7%) | 3 (1.3%) | 4 (1.7%) |
| White | 2,087 (79.6%) | 10,526 (82.5%) | 5,340 (86.9%) | 20,104 (84.8%) | 217 (73.3%) | 836 (64.0%) | 2,021 (79.3%) | 2,028 (83.3%) | 5,252 (86.9%) | 5,213 (86.3%) | 164 (71.3%) | 153 (64.8%) |
| Urban hospital (vs Rural) | 2,246 (85.7%) | 11,254 (88.2%) | 5,166 (84.1%) | 20,930 (88.3%) | 208 (70.3%) | 1,065 (81.5%) | 339 (13.3%) | 339 (13.9%) | 923 (15.3%) | 923 (15.3%) | 60 (26.1%) | 62 (26.3%) |
| Hospital Size |  |  |  |  |  |  |  |  |  |  |  |  |
| 000 to 099 | 364 (13.9%) | 1,403 (11.0%) | 966 (15.7%) | 2,324 (9.8%) | 63 (21.3%) | 191 (14.6%) | 342 (13.4%) | 323 (13.3%) | 947 (15.7%) | 947 (15.7%) | 44 (19.1%) | 44 (18.6%) |
| 100 to 199 | 548 (20.9%) | 2,118 (16.6%) | 1,314 (21.4%) | 4,304 (18.2%) | 63 (21.3%) | 185 (14.2%) | 531 (20.8%) | 531 (21.8%) | 1,273 (21.1%) | 1,273 (21.1%) | 44 (19.1%) | 44 (18.6%) |
| 200 to 299 | 535 (20.4%) | 2,902 (22.7%) | 1,327 (21.6%) | 5,717 (24.1%) | 53 (17.9%) | 362 (27.7%) | 529 (20.8%) | 502 (20.6%) | 1,316 (21.8%) | 1,316 (21.8%) | 45 (19.6%) | 49 (20.8%) |
| 300 to 399 | 510 (19.5%) | 2,885 (22.6%) | 1,307 (21.3%) | 4,717 (19.9%) | 35 (11.8%) | 178 (13.6%) | 504 (19.8%) | 494 (20.3%) | 1,298 (21.5%) | 1,298 (21.5%) | 30 (13.0%) | 30 (12.7%) |
| 400 to 499 | 448 (17.1%) | 1,266 (9.9%) | 786 (12.8%) | 2,856 (12.0%) | 44 (14.9%) | 226 (17.3%) | 425 (16.7%) | 421 (17.3%) | 765 (12.7%) | 765 (12.7%) | 35 (15.2%) | 35 (14.8%) |
| 500 and greater | 217 (8.3%) | 2,189 (17.2%) | 446 (7.3%) | 3,789 (16.0%) | 38 (12.8%) | 165 (12.6%) | 216 (8.5%) | 165 (6.8%) | 444 (7.3%) | 442 (7.3%) | 32 (13.9%) | 34 (14.4%) |

**Table S2: Comorbidity of Patients, by admission and payer type, before and after matching.**

|  | PreMatch | | | | | | Postmatch | | | | | |
| --- | --- | --- | --- | --- | --- | --- | --- | --- | --- | --- | --- | --- |
|  | Commercially-Insured | | Medicare | | Medicaid | | Commercially-Insured | | Medicare | | Medicaid | |
|  | Outpatient | Inpatient | Outpatient | Inpatient | Outpatient | Inpatient | Outpatient | Inpatient | Outpatient | Inpatient | Outpatient | Inpatient |
| **Elixhauser Index: mean (SD)** | 1.73 (1.35) | 1.88 (1.37) | 2.03 (1.50) | 2.34 (1.58) | 2.12 (1.51) | 2.30 (1.58) | 1.73 (1.32) | 1.74 (1.33) | 2.03 (1.48) | 2.06 (1.50) | 2.10 (1.38) | 2.13 (1.37) |
| **Elixhause Score Category** |  |  |  |  |  |  |  |  |  |  |  |  |
| 0 | 498 (19.0%) | 1,978 (15.5%) | 880 (14.3%) | 2,308 (9.7%) | 41 (13.9%) | 164 (12.5%) | 476 (18.7%) | 469 (19.3%) | 837 (13.9%) | 837 (13.9%) | 25 (10.9%) | 25 (10.6%) |
| 1 to 2 | 1,448 (55.2%) | 7,104 (55.7%) | 3,235 (52.6%) | 11,893 (50.2%) | 146 (49.3%) | 604 (46.2%) | 1,426 (56.0%) | 1,357 (55.7%) | 3,217 (53.2%) | 3,217 (53.3%) | 125 (54.3%) | 128 (54.2%) |
| 3 to 4 | 589 (22.5%) | 3,152 (24.7%) | 1,640 (26.7%) | 7,296 (30.8%) | 91 (30.7%) | 415 (31.8%) | 569 (22.3%) | 538 (22.1%) | 1,618 (26.8%) | 1,617 (26.8%) | 71 (30.9%) | 72 (30.5%) |
| 5 and above | 87 (3.3%) | 529 (4.1%) | 391 (6.4%) | 2,210 (9.3%) | 18 (6.1%) | 124 (9.5%) | 76 (3.0%) | 72 (3.0%) | 371 (6.1%) | 370 (6.1%) | 9 (3.9%) | 11 (4.7%) |
| **Individual Comorbidities** |  |  |  |  |  |  |  |  |  |  |  |  |
| Hypertension | 1,499 (57.2%) | 7,739 (60.6%) | 4,193 (68.2%) | 17,623 (74.3%) | 190 (64.2%) | 826 (63.2%) | 1,464 (57.5%) | 1,409 (57.8%) | 4,155 (68.8%) | 4,165 (68.9%) | 154 (67.0%) | 155 (65.7%) |
| Obesity | 907 (34.6%) | 4,654 (36.5%) | 1,617 (26.3%) | 6,504 (27.4%) | 109 (36.8%) | 469 (35.9%) | 886 (34.8%) | 771 (31.7%) | 1,579 (26.1%) | 1,481 (24.5%) | 85 (37.0%) | 81 (34.3%) |
| Diabetes | 420 (16.0%) | 2,440 (19.1%) | 1,252 (20.4%) | 5,832 (24.6%) | 67 (22.6%) | 328 (25.1%) | 406 (15.9%) | 424 (17.4%) | 1,230 (20.4%) | 1,315 (21.8%) | 53 (23.0%) | 59 (25.0%) |
| Hypothyroidism | 375 (14.3%) | 1,811 (14.2%) | 1,031 (16.8%) | 4,613 (19.5%) | 35 (11.8%) | 162 (12.4%) | 359 (14.1%) | 342 (14.0%) | 1,017 (16.8%) | 1,029 (17.0%) | 25 (10.9%) | 24 (10.2%) |
| Chronic Pulmonary Disease | 308 (11.7%) | 1,711 (13.4%) | 907 (14.8%) | 3,979 (16.8%) | 66 (22.3%) | 351 (26.9%) | 298 (11.7%) | 305 (12.5%) | 881 (14.6%) | 874 (14.5%) | 51 (22.2%) | 58 (24.6%) |
| Depression | 343 (13.1%) | 1,865 (14.6%) | 795 (12.9%) | 3,619 (15.3%) | 59 (19.9%) | 291 (22.3%) | 328 (12.9%) | 327 (13.4%) | 777 (12.9%) | 826 (13.7%) | 46 (20.0%) | 47 (19.9%) |
| Cardiac Arrhythmia | 161 (6.1%) | 763 (6.0%) | 646 (10.5%) | 3,154 (13.3%) | 14 (4.7%) | 67 (5.1%) | 156 (6.1%) | 118 (4.8%) | 641 (10.6%) | 675 (11.2%) | 12 (5.2%) | 6 (2.5%) |
| Renal failure | 99 (3.8%) | 451 (3.5%) | 491 (8.0%) | 2,371 (10.0%) | 20 (6.8%) | 82 (6.3%) | 83 (3.3%) | 81 (3.3%) | 478 (7.9%) | 487 (8.1%) | 11 (4.8%) | 8 (3.4%) |
| Rheumatoid Arthritis collagen | 91 (3.5%) | 530 (4.2%) | 273 (4.4%) | 1,215 (5.1%) | 9 (3.0%) | 60 (4.6%) | 88 (3.5%) | 94 (3.9%) | 268 (4.4%) | 288 (4.8%) | 8 (3.5%) | 11 (4.7%) |
| Congestive Heart Failure | 55 (2.1%) | 248 (1.9%) | 253 (4.1%) | 1,202 (5.1%) | 15 (5.1%) | 40 (3.1%) | 52 (2.0%) | 43 (1.8%) | 250 (4.1%) | 214 (3.5%) | 12 (5.2%) | 8 (3.4%) |
| Valvular disease | 43 (1.6%) | 264 (2.1%) | 178 (2.9%) | 1,102 (4.6%) | 4 (1.4%) | 22 (1.7%) | 40 (1.6%) | 49 (2.0%) | 176 (2.9%) | 239 (4.0%) | 3 (1.3%) | 3 (1.3%) |
| Peripheral Vascular Disease | 31 (1.2%) | 182 (1.4%) | 165 (2.7%) | 793 (3.3%) | 5 (1.7%) | 26 (2.0%) | 30 (1.2%) | 34 (1.4%) | 163 (2.7%) | 149 (2.5%) | 3 (1.3%) | 4 (1.7%) |
| Fluid and electrolyte disorders | 45 (1.7%) | 284 (2.2%) | 143 (2.3%) | 750 (3.2%) | 5 (1.7%) | 53 (4.1%) | 42 (1.6%) | 59 (2.4%) | 140 (2.3%) | 149 (2.5%) | 4 (1.7%) | 5 (2.1%) |
| Other neurological disorders | 27 (1.0%) | 189 (1.5%) | 123 (2.0%) | 584 (2.5%) | 5 (1.7%) | 35 (2.7%) | 26 (1.0%) | 31 (1.3%) | 118 (2.0%) | 132 (2.2%) | 1 (0.4%) | 7 (3.0%) |
| Liver disease | 40 (1.5%) | 151 (1.2%) | 78 (1.3%) | 309 (1.3%) | 4 (1.4%) | 44 (3.4%) | 39 (1.5%) | 34 (1.4%) | 74 (1.2%) | 71 (1.2%) | 3 (1.3%) | 6 (2.5%) |
| Coagulopathy | 31 (1.2%) | 196 (1.5%) | 66 (1.1%) | 423 (1.8%) | 1 (0.3%) | 21 (1.6%) | 30 (1.2%) | 24 (1.0%) | 64 (1.1%) | 88 (1.5%) | 1 (0.4%) | 3 (1.3%) |
| Deficiency anemia | 22 (0.8%) | 149 (1.2%) | 62 (1.0%) | 346 (1.5%) | 6 (2.0%) | 21 (1.6%) | 18 (0.7%) | 27 (1.1%) | 62 (1.0%) | 66 (1.1%) | 3 (1.3%) | 5 (2.1%) |
| Pulmonary circulation disorders | 7 (0.3%) | 47 (0.4%) | 42 (0.7%) | 233 (1.0%) | 2 (0.7%) | 6 (0.5%) | 7 (0.3%) | 9 (0.4%) | 41 (0.7%) | 55 (0.9%) | 2 (0.9%) | 0 (0.0%) |
| Alcohol abuse | 19 (0.7%) | 105 (0.8%) | 33 (0.5%) | 161 (0.7%) | 4 (1.4%) | 31 (2.4%) | 19 (0.7%) | 17 (0.7%) | 31 (0.5%) | 27 (0.4%) | 3 (1.3%) | 4 (1.7%) |
| Drug abuse | 21 (0.8%) | 138 (1.1%) | 29 (0.5%) | 258 (1.1%) | 5 (1.7%) | 48 (3.7%) | 20 (0.8%) | 20 (0.8%) | 28 (0.5%) | 47 (0.8%) | 2 (0.9%) | 6 (2.5%) |
| Psychoses | 0 (0.0%) | 6 (0.0%) | 8 (0.1%) | 52 (0.2%) | 2 (0.7%) | 11 (0.8%) | 0 (0.0%) | 3 (0.1%) | 7 (0.1%) | 14 (0.2%) | 1 (0.4%) | 0 (0.0%) |

**Table S3: Hospital costs by patient payer type.**

|  |  |  |  |  |
| --- | --- | --- | --- | --- |
|  |  | Commercial | Medicare | Medicaid |
| **Index Costs** | Outpatient | $ 14,805 (95%CI:$ 14,433-$ 15,176) | $ 14,788 (95%CI:$ 14,519-$ 15,058) | $ 15,102 (95%CI:$ 13,980-$ 16,225) |
|  | Inpatient | $ 17,255 (95%CI:$ 16,820-$ 17,690) | $ 17,236 (95%CI:$ 16,920-$ 17,553) | $ 17,602 (95%CI:$ 16,296-$ 18,908) |
| **90-Day Knee Related Costs*** | Outpatient | $ 14,920 (95%CI:$ 14,568-$ 15,273) | $ 15,140 (95%CI:$ 14,877-$ 15,403) | $ 15,661 (95%CI:$ 14,569-$ 16,753) |
|  | Inpatient | $ 17,686 (95%CI:$ 17,264-$ 18,108) | $ 17,947 (95%CI:$ 17,634-$ 18,260) | $ 18,564 (95%CI:$ 17,271-$ 19,857) |
| **90-Day All-Cause Costs*** | Outpatient | $ 15,411 (95%CI:$ 15,002-$ 15,821) | $ 15,709 (95%CI:$ 15,406-$ 16,012) | $ 16,436 (95%CI:$ 15,146-$ 17,726) |
|  | Inpatient | $ 18,224 (95%CI:$ 17,739-$ 18,710) | $ 18,576 (95%CI:$ 18,215-$ 18,937) | $ 19,436 (95%CI:$ 17,912-$ 20,959) |

**Includes index costs.*

*CI: Confidence Interval. TKA: Total Knee Arthroplasty.*

**Table S4: Exploratory outcomes, for Commercially-insured and Medicare Patients.**

|  | Commercially Insured | | Medicare | |  |
| --- | --- | --- | --- | --- | --- |
|  | Outpatient | Inpatient | Outpatient | Inpatient | P value |
| **N** | 2,547 | 2,436 | 6,043 | 6,041 |  |
| **Length of Stay: mean (SD)** | 0.01 (0.30) | 1.85 (0.95) | 0.00 (0.00) | 2.17 (1.25) | <0.001 |
| **Discharge Status** |  |  |  |  | <0.001 |
| Discharged to HHO | 461 (18%) | 961 (39%) | 1,074 (18%) | 2,223 (37%) |  |
| Home | 2,064 (81%) | 1,324 (54%) | 4,749 (79%) | 2,657 (44%) |  |
| SNF Other | 22 (1%) | 151 (6%) | 220 (4%) | 1,161 (19%) |  |
| **Operating Room Time: mean minutes (SD)** | 134.79 (37.35) | 135.41 (35.48) | 130.57 (32.29) | 132.71 (34.53) | <0.001 |
| **Mean Costs (SD)** |  |  |  |  |  |
| Index costs | 14,674.98 (5,588.99) | 16,928.94 (22,911.34) | 14,928.86 (5,282.11) | 17,215.34 (12,873.49) | <0.001 |
| 90-day all-cause costs | 15,331.40 (6,153.25) | 17,816.36 (23,454.07) | 15,808.65 (7,172.19) | 18,582.57 (14,761.98) | <0.001 |
| 90-day knee-related costs | 14,909.19 (5,854.61) | 17,292.97 (23,170.27) | 15,269.49 (6,064.88) | 17,876.90 (14,002.74) | <0.001 |
| **Reoperation at 90 Days** | 14 (0.55%) | 17 (0.70%) | 32 (0.53%) | 42 (0.70%) | 0.058 |
| **All Cause Readmission at 90 Days** | 697 (27%) | 715 (29%) | 1,669 (28%) | 1,957 (32%) | <0.001 |

Table S5: Model estimates on increased index hospital costs as a function of patient and provider variables.

| **Variables** | **Estimate** | **Lower 2.5%** | **Upper 97.5%** | **P Value** |  |
| --- | --- | --- | --- | --- | --- |
| (Intercept) | 12237.99 | 11557.94 | 12958.04 | 0.00 |  |
| Inpatient (vs Outpatient) | 1.17 | 1.14 | 1.19 | 0.00 |  |
| ***Payer: Reference = Commercial*** | | | | |  |
| Medicare | 1.00 | 0.97 | 1.03 | 0.93 |  |
| Medicaid | 1.02 | 0.94 | 1.10 | 0.61 |  |
| ***Hospital size: Reference = 500+ Beds*** | | | | |  |
| 000-099 | 1.06 | 1.00 | 1.13 | 0.04 |  |
| 100-199 | 1.10 | 1.04 | 1.16 | 0.00 |  |
| 200-299 | 1.05 | 0.99 | 1.10 | 0.08 |  |
| 300-399 | 0.98 | 0.93 | 1.03 | 0.35 |  |
| 400-499 | 0.95 | 0.90 | 1.01 | 0.10 |  |
| **Rural Hospital (vs Urban)** | 1.02 | 0.99 | 1.06 | 0.23 |  |
| ***Geographic Location: Reference = East North Central**** | | | | |  |
| East South Central | 1.05 | 1.00 | 1.09 | 0.04 |  |
| Middle Atlantic | 0.98 | 0.93 | 1.03 | 0.43 |  |
| Mountain | 0.92 | 0.77 | 1.09 | 0.33 |  |
| New England | 1.14 | 1.04 | 1.24 | 0.01 |  |
| Pacific | 1.37 | 1.31 | 1.43 | 0.00 |  |
| South Atlantic | 1.35 | 1.30 | 1.41 | 0.00 |  |
| West North Central | 1.00 | 0.95 | 1.06 | 0.92 |  |
| West South Central | 1.22 | 1.14 | 1.31 | 0.00 |  |
| **Sex: Male (vs female)** | 1.00 | 0.97 | 1.02 | 0.69 |  |
| ***Race: Reference = White*** | | | | |  |
| Black | 1.07 | 1.03 | 1.12 | 0.00 |  |
| Asian | 1.02 | 0.91 | 1.15 | 0.70 |  |
|  |  |  |  |  |  |
| *Geographic divisions: https://www.cdc.gov/nchs/hus/sources-definitions/geographic-region.htm | | | | | |

Table S6: Model estimates on increased 90-day all-cause hospital costs as a function of patient and provider variables.

| **Variables** | **Estimate** | **Lower 2.5%** | **Upper 97.5%** | **P Value** |
| --- | --- | --- | --- | --- |
| (Intercept) | 13407.06 | 12620.39 | 14242.78 | 0.00 |
| Inpatient (vs Outpatient) | 1.18 | 1.15 | 1.21 | 0.00 |
| ***Payer: Reference = Commercial*** | | | | |
| Medicare | 1.02 | 0.99 | 1.05 | 0.18 |
| Medicaid | 1.07 | 0.98 | 1.16 | 0.12 |
| ***Hospital size: Reference = 500+ Beds*** | | | | |
| 000-099 | 1.03 | 0.97 | 1.10 | 0.30 |
| 100-199 | 1.08 | 1.02 | 1.14 | 0.01 |
| 200-299 | 1.02 | 0.96 | 1.07 | 0.55 |
| 300-399 | 0.96 | 0.91 | 1.01 | 0.12 |
| 400-499 | 0.94 | 0.88 | 0.99 | 0.03 |
| **Rural Hospital (vs Urban)** | 1.06 | 1.02 | 1.10 | 0.00 |
| ***Geographic Location: Reference = East North Central**** | | | | |
| East South Central | 1.00 | 0.95 | 1.04 | 0.83 |
| Middle Atlantic | 0.94 | 0.89 | 1.00 | 0.03 |
| Mountain | 1.08 | 0.90 | 1.30 | 0.41 |
| New England | 1.07 | 0.97 | 1.18 | 0.16 |
| Pacific | 1.32 | 1.25 | 1.38 | 0.00 |
| South Atlantic | 1.30 | 1.25 | 1.36 | 0.00 |
| West North Central | 0.96 | 0.91 | 1.02 | 0.21 |
| West South Central | 1.14 | 1.06 | 1.22 | 0.00 |
| **Gender: Male (vs female)** | 1.00 | 0.97 | 1.02 | 0.93 |
| ***Race: Reference = White*** | | | | |
| Black | 1.08 | 1.03 | 1.13 | 0.00 |
| Asian | 1.01 | 0.89 | 1.15 | 0.85 |
|  |  |  |  |  |
| *Geographic divisions: https://www.cdc.gov/nchs/hus/sources-definitions/geographic-region.htm | | | | |

Table S7: Model estimates on increased 90-day knee-related hospital costs as a function of patient and provider variables.

| **Variables** | **Estimate** | **Lower 2.5%** | **Upper 97.5%** | **P Value** |  |  |
| --- | --- | --- | --- | --- | --- | --- |
| (Intercept) | 12905.97 | 12230.04 | 13619.26 | 0.00 |  |  |
| Inpatient (vs Outpatient) | 1.19 | 1.16 | 1.21 | 0.00 |  |  |
| ***Payer: Reference = Commercial*** | | | | |  |  |
| Medicare | 1.01 | 0.99 | 1.04 | 0.25 |  |  |
| Medicaid | 1.05 | 0.98 | 1.13 | 0.19 |  |  |
| ***Hospital size: Reference = 500+ Beds*** | | | | |  |  |
| 000-099 | 1.03 | 0.97 | 1.08 | 0.35 |  |  |
| 100-199 | 1.08 | 1.03 | 1.13 | 0.00 |  |  |
| 200-299 | 1.02 | 0.97 | 1.07 | 0.49 |  |  |
| 300-399 | 0.96 | 0.92 | 1.00 | 0.07 |  |  |
| 400-499 | 0.89 | 0.85 | 0.94 | 0.00 |  |  |
| **Rural Hospital (vs Urban)** | 1.05 | 1.02 | 1.09 | 0.00 |  |  |
| ***Geographic Location: Reference = East North Central*** | | | | |  |  |
| East South Central | 1.02 | 0.91 | 1.14 | 0.76 |  |  |
| Middle Atlantic | 1.03 | 0.99 | 1.08 | 0.15 |  |  |
| Mountain | 1.01 | 0.98 | 1.06 | 0.47 |  |  |
| New England | 0.99 | 0.95 | 1.04 | 0.81 |  |  |
| Pacific | 0.95 | 0.81 | 1.11 | 0.49 |  |  |
| South Atlantic | 1.10 | 1.01 | 1.20 | 0.02 |  |  |
| West North Central | 1.34 | 1.29 | 1.40 | 0.00 |  |  |
| West South Central | 1.30 | 1.26 | 1.35 | 0.00 |  |  |
| **Gender: Male (vs female)** | 0.98 | 0.92 | 1.04 | 0.49 |  |  |
| ***Race: Reference = White*** | | | | |  |  |
| Black | 0.99 | 0.97 | 1.01 | 0.43 |  |  |
| Asian | 1.08 | 1.04 | 1.13 | 0.00 |  |  |
|  |  |  |  |  |  |  |
| Geographic divisions: https://www.cdc.gov/nchs/hus/sources-definitions/geographic-region.htm | | | | | | |

Table S8: Odds of discharge to a skilled nursing facility, as a function of patient and provider variables.

| **Variables** | **Estimate** | **Lower 2.5%** | **Upper 97.5%** | **P Value** |  |
| --- | --- | --- | --- | --- | --- |
| (Intercept) | 0.97 | 0.95 | 0.99 | 0.00 |  |
| Inpatient (vs Outpatient) | 1.13 | 1.12 | 1.14 | 0.00 |  |
| ***Payer: Reference = Commercial*** | | | | |  |
| Medicare | 1.08 | 1.07 | 1.09 | 0.00 |  |
| Medicaid | 1.00 | 0.97 | 1.02 | 0.82 |  |
| ***Hospital size: Reference = 500+ Beds*** | | | | |  |
| 000-099 | 1.03 | 1.01 | 1.06 | 0.00 |  |
| 100-199 | 1.04 | 1.02 | 1.06 | 0.00 |  |
| 200-299 | 1.04 | 1.02 | 1.06 | 0.00 |  |
| 300-399 | 1.00 | 0.98 | 1.02 | 0.96 |  |
| 400-499 | 0.99 | 0.97 | 1.01 | 0.33 |  |
| **Rural Hospital (vs Urban)** | 1.01 | 0.99 | 1.02 | 0.24 |  |
| ***Geographic Location: Reference = East North Central*** | | | | |  |
| East South Central | 1.00 | 0.99 | 1.02 | 0.74 |  |
| Middle Atlantic | 1.08 | 1.06 | 1.10 | 0.00 |  |
| Mountain | 0.93 | 0.87 | 0.99 | 0.02 |  |
| New England | 0.94 | 0.91 | 0.97 | 0.00 |  |
| Pacific | 0.98 | 0.96 | 0.99 | 0.00 |  |
| South Atlantic | 0.96 | 0.94 | 0.97 | 0.00 |  |
| West North Central | 0.96 | 0.94 | 0.98 | 0.00 |  |
| West South Central | 0.99 | 0.97 | 1.02 | 0.68 |  |
| **Gender: Male (vs female)** | 0.97 | 0.96 | 0.98 | 0.00 |  |
| ***Race: Reference = White*** | | | | |  |
| Black | 1.04 | 1.02 | 1.06 | 0.00 |  |
| Asian | 1.01 | 0.97 | 1.06 | 0.62 |  |
|  |  |  |  |  |  |
| Geographic divisions: https://www.cdc.gov/nchs/hus/sources-definitions/geographic-region.htm | | | | | |

**Table S9: Odds ratios or reoperation, as a function of patient and provider characteristics.**

| **Variables** | **Estimate** | **Lower 2.5%** | **Upper 97.5%** | **P Value** |
| --- | --- | --- | --- | --- |
| (Intercept) | 1.00 | 1.00 | 1.01 | 0.15 |
| Inpatient (vs Outpatient) | 1.00 | 1.00 | 1.00 | 0.53 |
| ***Payer: Reference = Commercial*** | | | | |
| Medicare | 1.00 | 1.00 | 1.00 | 0.63 |
| Medicaid | 1.01 | 1.00 | 1.02 | 0.02 |
| ***Hospital size: Reference = 500+ Beds*** | | | | |
| 000-099 | 1.00 | 1.00 | 1.01 | 0.64 |
| 100-199 | 1.00 | 1.00 | 1.01 | 0.16 |
| 200-299 | 1.00 | 1.00 | 1.01 | 0.10 |
| 300-399 | 1.00 | 1.00 | 1.01 | 0.33 |
| 400-499 | 1.00 | 1.00 | 1.01 | 0.33 |
| **Rural Hospital (vs Urban)** | 1.00 | 1.00 | 1.00 | 0.93 |
| ***Geographic Location: Reference = East North Central*** | | | | |
| East South Central | 1.00 | 0.99 | 1.00 | 0.06 |
| Middle Atlantic | 1.00 | 1.00 | 1.01 | 0.76 |
| Mountain | 0.99 | 0.97 | 1.01 | 0.30 |
| New England | 0.99 | 0.98 | 1.00 | 0.05 |
| Pacific | 0.99 | 0.99 | 1.00 | 0.02 |
| South Atlantic | 1.00 | 1.00 | 1.00 | 0.90 |
| West North Central | 1.00 | 0.99 | 1.00 | 0.27 |
| West South Central | 1.00 | 0.99 | 1.00 | 0.19 |
| **Gender: Male (vs female)** | 1.00 | 1.00 | 1.00 | 0.66 |
| ***Race: Reference = White*** | | | | |
| Black | 1.00 | 0.99 | 1.00 | 0.45 |
| Asian | 1.00 | 0.99 | 1.01 | 0.94 |
|  |  |  |  |  |
| Geographic divisions: https://www.cdc.gov/nchs/hus/sources-definitions/geographic-region.htm | | | | |

**Table S10: Odds ratios of all-cause readmission, as a function of patient and provider characteristics.**

| **Variables** | **Estimate** | **Lower 2.5%** | **Upper 97.5%** | **P Value** |
| --- | --- | --- | --- | --- |
| (Intercept) | 1.42 | 1.38 | 1.47 | 0.00 |
| Inpatient (vs Outpatient) | 1.05 | 1.03 | 1.06 | 0.00 |
| ***Payer: Reference = Commercial*** | | | | |
| Medicare | 1.01 | 1.00 | 1.03 | 0.15 |
| Medicaid | 1.15 | 1.10 | 1.20 | 0.00 |
| ***Hospital size: Reference = 500+ Beds*** | | | | |
| 000-099 | 1.05 | 1.01 | 1.09 | 0.01 |
| 100-199 | 1.05 | 1.02 | 1.08 | 0.00 |
| 200-299 | 1.00 | 0.97 | 1.03 | 0.89 |
| 300-399 | 1.00 | 0.97 | 1.03 | 0.87 |
| 400-499 | 0.95 | 0.92 | 0.98 | 0.00 |
| **Rural Hospital (vs Urban)** | 1.17 | 1.15 | 1.20 | 0.00 |
| ***Geographic Location: Reference = East North Central*** | | | | |
| East South Central | 0.87 | 0.85 | 0.89 | 0.00 |
| Middle Atlantic | 0.84 | 0.81 | 0.86 | 0.00 |
| Mountain | 0.81 | 0.73 | 0.89 | 0.00 |
| New England | 0.78 | 0.74 | 0.82 | 0.00 |
| Pacific | 0.90 | 0.88 | 0.93 | 0.00 |
| South Atlantic | 0.91 | 0.89 | 0.93 | 0.00 |
| West North Central | 0.81 | 0.79 | 0.84 | 0.00 |
| West South Central | 0.70 | 0.67 | 0.73 | 0.00 |
| **Gender: Male (vs female)** | 1.00 | 0.99 | 1.02 | 0.67 |
| ***Race: Reference = White*** | | | | |
| Black | 1.05 | 1.02 | 1.08 | 0.00 |
| Asian | 0.95 | 0.88 | 1.02 | 0.13 |
|  |  |  |  |  |
| Geographic divisions: https://www.cdc.gov/nchs/hus/sources-definitions/geographic-region.htm | | | | |
